# An assessment of uranium in groundwater in the Grand Canyon region

**DOI:** 10.1038/s41598-021-01621-8

**Published:** 2021-11-16

**Authors:** Fred D Tillman, Kimberly R. Beisner, Jessica R. Anderson, Joel A. Unema

**Affiliations:** 1 Arizona Water Science Center, U.S. Geological Survey, Tucson, AZ USA; 2 New Mexico Water Science Center, U.S. Geological Survey, Albuquerque, NM USA; 3 Arizona Water Science Center, U.S. Geological Survey, Flagstaff, AZ USA

**Keywords:** Environmental sciences, Hydrology

## Abstract

The Grand Canyon region in northern Arizona is a home or sacred place of origin for many Native Americans and is visited by over 6 million tourists each year. Most communities in the area depend upon groundwater for all water uses. Some of the highest-grade uranium ore in the United States also is found in the Grand Canyon region. A withdrawal of over 4000 km^2^ of Federal land in the Grand Canyon region from new uranium mining activities for 20 years was instituted in 2012, owing in part to a lack of scientific data on potential effects from uranium mining on water resources in the area. The U.S. Geological Survey has collected groundwater chemistry samples since 1981 in the Grand Canyon region to better understand the current state of groundwater quality, to monitor for changes in groundwater quality that may be the result of mining activities, and to identify "hot spots" with elevated metal concentrations and investigate the causes. This manuscript presents results for the assessment of uranium in groundwater in the Grand Canyon region. Analytical results for uranium in groundwater in the Grand Canyon region were available for 573 samples collected from 180 spring sites and 26 wells from September 1, 1981 to October 7, 2020. Samples were collected from springs issuing from stratigraphic units above, within, and below the Permian strata that host uranium ore in breccia pipes in the area. Maximum uranium concentrations at groundwater sites in the region ranged from less than 1 µg/L at 23 sites (11%) to 100 µg/L or more at 4 sites (2%). Of the 206 groundwater sites sampled, 195 sites (95%) had maximum observed uranium concentrations less than the U.S. Environmental Protection Agency’s Maximum Contaminant Level of 30 µg/L for drinking water and 177 sites (86%) had uranium concentrations less than the 15 µg/L Canadian benchmark for protection of aquatic life in freshwater. The establishment of baseline groundwater quality is an important first step in monitoring for change in water chemistry throughout mining lifecycles and beyond to ensure the health of these critical groundwater resources.

## Introduction

**Figure 1:**
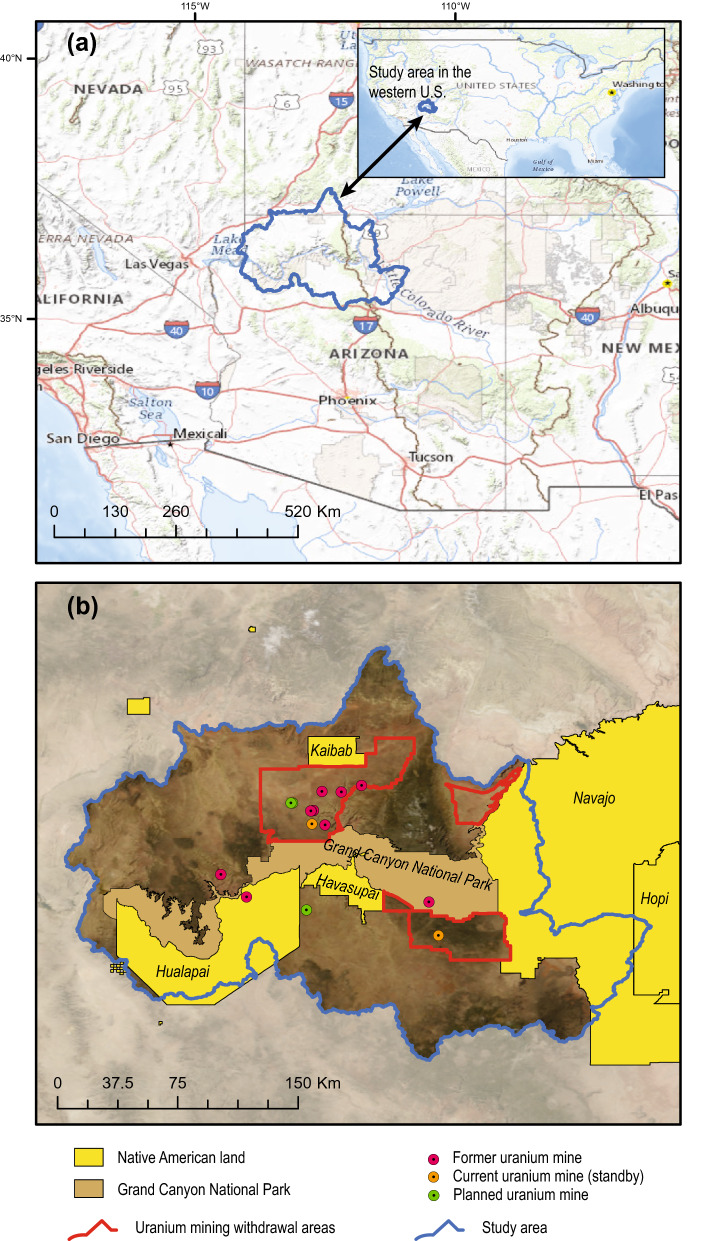
Map of study area within the western United States (**a**) and closeup of study area (**b**) showing locations of Tribal Nations, uranium mines, Grand Canyon National Park, and uranium mining withdrawal areas. Maps created in Esri ArcMap 10.8.1 (https://www.esri.com/). Basemap images from the public domain USGS National Map^11^.

The Grand Canyon in northern Arizona is an international tourist destination, a United Nations World Heritage Site^1^, and a home or sacred place of origin for many Native Americans (Fig. 1). Although the Colorado River, a primary source of drinking and irrigation water for 35 million people in the United States and Mexico^2^, runs through the region, most communities in the area do not have rights or access to water from the river and are entirely dependent upon groundwater for all water uses^3–5^. These groundwater-dependent communities include the Havasupai Nation, the Hualapai Nation, the towns of Tusayan, Williams, and Jacobs Lake, and even Grand Canyon National Park, which is visited by as many as 6 million people each year^6^. Additionally, groundwater discharge at spring sites sustains important ecosystems in the area^7–10^.

Some of the highest-grade uranium ore in the United States is found in the Grand Canyon region^12^. In 2012, then-U.S. Secretary of the Interior Ken Salazar withdrew over 4000 km^2^ (1 million acres) of Federal land in the Grand Canyon region from new uranium mining activities for the next 20 years, subject to valid existing rights^13^. Lack of scientific data on potential effects of uranium mining activities on cultural, biological, and water resources in the area was a key factor in limiting future uranium mining. Several water-resources investigations have been conducted in the region^14–19^. Since 2014, the U.S. Geological Survey (USGS) has planned and conducted scientific investigations on potential effects from uranium mining in the area^20–24^. Among other activities, USGS collects groundwater samples in the Grand Canyon region to understand the current state of groundwater quality, to monitor for changes in groundwater quality that may be the result of mining activities, to identify "hot spots" with elevated metal concentrations, and to investigate the causes of elevated metal concentrations. This manuscript presents results for the assessment of uranium in groundwater in the Grand Canyon region through 2020.

### Study area

**Figure 2:**
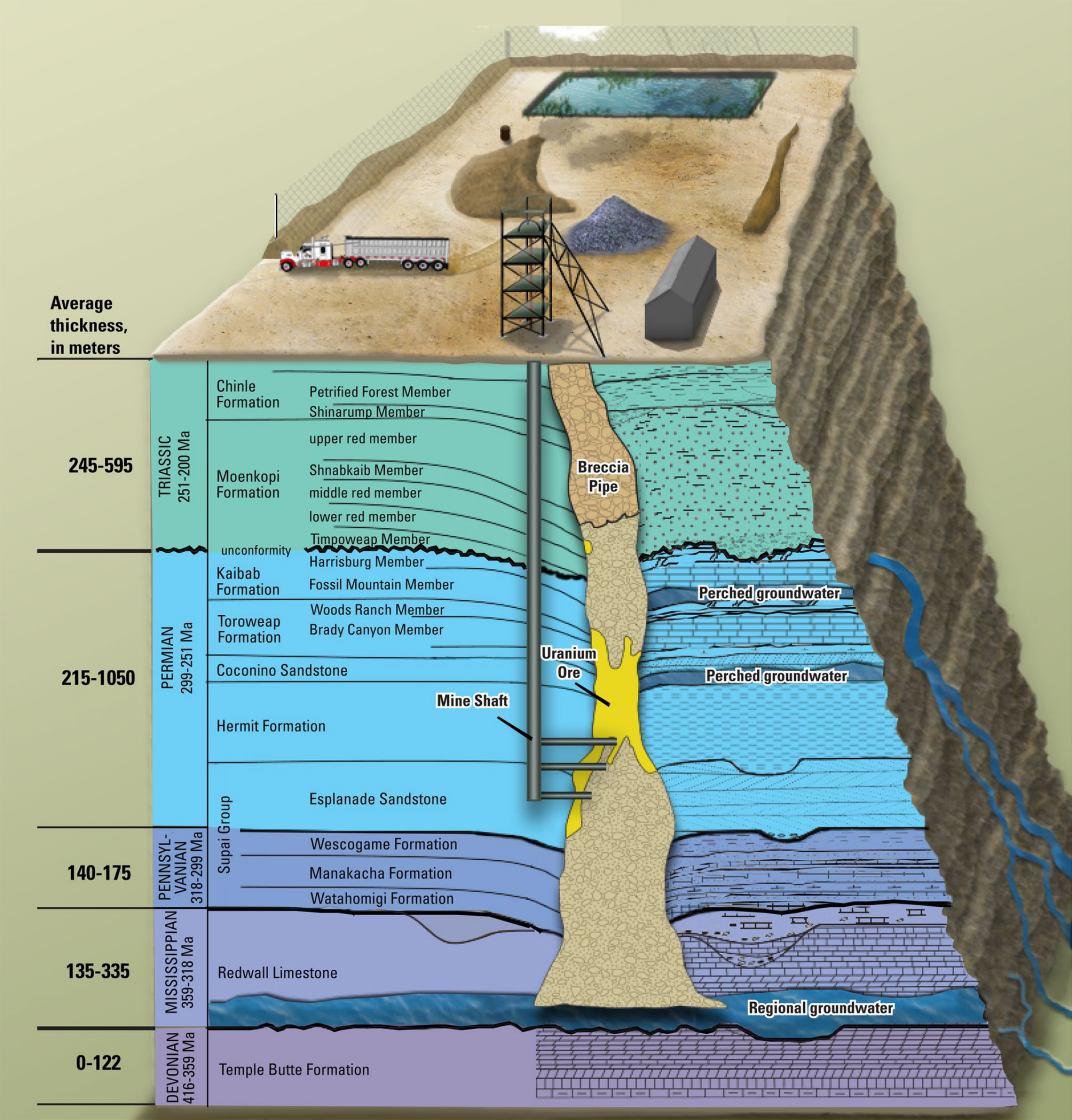
Schematic of lithology in the Grand Canyon area related to breccia pipe uranium mining. Adapted from U.S. Geological Survey^27^.

Groundwater in the Grand Canyon area is present in a shallower perched system and a deeper regional groundwater system. Perched groundwater is discontinuous throughout the area, but where present, is located about 300 m below plateau land surface in the Permian-age Coconino Sandstone (Fig. 2). The regional aquifer is > 1000 m below the plateau surface in the Mississippian-age Redwall Limestone, the Devonian-age Temple Butte Formation, and the underlying Cambrian-age Muav Limestone of the Tonto Group (Fig. 2). The regional groundwater system, also known as the Redwall–Muav aquifer, is present throughout the Grand Canyon region except within canyons where the aquifer units have been eroded away. The age of water in the perched and regional groundwater systems varies greatly in the area. At some spring sites along the Kaibab Plateau, only days to months pass between water recharging the groundwater system and discharging at the springs^19,25^. At other well and spring sites in the region, groundwater is thousands of years old^15,26^. Owing to the remoteness of the area and depth to groundwater, few wells are available with which to delineate groundwater basins and flow paths. For this study, USGS 8- and 10- digit Hydrologic Unit Code (HUC) basins are used as a proxy for groundwater basin boundaries (Fig. 1). Study area boundaries are composed of topographic watershed boundaries that drain to the Colorado River between Lees Ferry in the east and the Grand Wash Cliffs (near the eastern edge of Lake Mead) in the west (Fig. 1).

Uranium ore in the Grand Canyon area is hosted in collapse features known as breccia pipes, which are chimney-like formations filled with rubble (breccia). The ore zones are found mainly at the levels of the Permian-age stratigraphic units (Fig. 2), primarily at the horizons of the Coconino Sandstone, Hermit Formation, and the Esplanade Sandstone. There are thousands of collapse features in the Grand Canyon area but only a relative few are known to be mineralized with uranium ore^28^. Uranium mining in the Grand Canyon area began in the early 1950s at the Orphan copper mine, within a breccia pipe on the South Rim of Grand Canyon^29^. Presently, there are 11 former and 2 current (in standby status as of 2020) breccia pipe uranium mines in the Grand Canyon region. Two additional breccia pipe uranium mines currently being planned are the EZ Mine complex on Federal land and the Wate Pipe outside the Federal withdrawal area (Fig. 1). Breccia pipe uranium mines in the Grand Canyon region may encounter perched groundwater when mining shafts are installed, but the bottom of mine shafts are often hundreds of meters above the regional Redwall–Muav aquifer (Fig. 2). In addition to uranium, other trace elements may be enriched in mineralized breccia pipes including silver, arsenic, barium, cadmium, cobalt, copper, mercury, molybdenum, nickel, lead, antimony, strontium, vanadium, and zinc^30^. Many of these elements also occur in rock units and surface soils in the area, but at substantially lower concentrations^31^.

Solution-collapse breccia pipes in the southwestern United States are unique in the world for uranium deposits and have a unique International Atomic Energy Agency uranium deposit classification category^32^. Petrographic studies indicate that the uranium mineralization in these breccia pipes occurred late in the paragenetic sequence, after most of the base metal sulfides were deposited. Thus, the reductant for the precipitation of uranium oxide in the breccia pipes is interpreted to be pre-existing sulfide minerals. In contrast, the reductants for the stratabound uranium deposits more commonly found throughout the Colorado Plateau^33^ are usually interpreted to be organic matter and/or hydrocarbons^34^. In contrast to the stratabound uranium deposits in the region, breccia pipe uranium deposits also contain a large, diverse suite of sulfide minerals. Throughout the Colorado Plateau surrounding the Grand Canyon region, there are elevated concentrations of uranium, vanadium, and copper, along with other trace elements associated with stratabound roll front (sandstone type) and tabular mineralized deposits, in the Morrison, Chinle, and Cutler Formations^35–38^. Elevated concentrations of trace elements in water resources associated with these deposits occur throughout the region and are distinct from the breccia pipe uranium deposits.

While uranium (^238^U) exhibits radioactivity, its half-life of 4.5 billion years results in relatively low carcinogenicity^39,40^. Consuming water containing uranium for extended periods of time, however, has been shown to have negative effects on kidney function^41,42^. Other metals commonly associated with mineralized breccia pipes may pose a greater risk to human health than uranium (Supplementary Fig. S1). For example, arsenic has a U.S. Environmental Protection Agency (USEPA) allowable drinking water concentration (Maximum Contaminant Level or MCL) of 10 µg/L, which is one-third of the 30 µg/L allowable drinking water concentration for uranium^43^. This manuscript, however, focuses on the presence of uranium in groundwater in the Grand Canyon region as uranium has been shown to be a tracer of ore-derived material dispersion from breccia pipe mines to nearby soils^44^ and is of particular concern to residents of and visitors to the area.

### Sources and mobility of uranium in groundwater

In the United States, a USGS study detected uranium (reporting limits of 0.003–1 µg/L) in 35% of wells in aquifers used for drinking water but found uranium concentrations in excess of the USEPA MCL of 30 µg/L in only 1.6% of samples nationally^45^. In the southwestern United States, 7% of the drinking water wells sampled exceeded the MCL^46^. A natural source of uranium in groundwater in the Southwest is the weathering of metamorphic and granitic rocks and sediment derived from these rocks^46^. A recent USGS study of element concentrations from more than 700 surface soil samples in the Grand Canyon region indicated a median uranium concentration of 3.5 mg/kg and a maximum concentration of 8.8 mg/kg^31^. Additionally, the mining of uranium deposits may expose ore and waste rock to water that can dissolve or desorb uranium from solids and then be transported to aquifer systems.

The mobility of uranium in groundwater is dependent on a complex interaction of hydrogen ion concentration (pH), redox conditions, the availability of complexing ions, and the presence of highly sorptive materials^47–49^ (Supplementary Fig. S2). Uranium is generally mobile in groundwater under acidic (pH < 5) conditions in oxidizing environments and relatively immobile at circumneutral pH values under anoxic conditions^48,50,51^. Under oxidizing conditions, however, uranium is present as the oxycation uranyl (UO_2_^2+^), which readily forms stable uranyl aqueous complexes in the presence of dissolved carbonate, sulfate, and phosphate species. Uranyl carbonate and phosphate complexes greatly increase the mobility of uranium, extending solubility through a higher range of pH values (from 5 to 12)^48^ and more reducing redox states^50^. However, concentrations of neutral and negatively charged uranium complexes may be diminished at pHs > 5 in oxidized groundwater in the presence of clays, zeolites, and hydrous metal oxides, such as hydrous ferric oxide, which have highly sorptive surfaces^47^.

Human activities may increase the otherwise naturally occurring concentrations of uranium in groundwater. Irrigation water high in bicarbonate (HCO_3_^−^) has been shown to leach uranium from sediments in some agricultural areas of California’s Central Valley, resulting in elevated uranium (as high as 500 µg/L) in groundwater wells^40,52^. Wells screened over multiple aquifer units in the High Plains aquifer have been implicated in elevated uranium concentrations whereby the pumping of the deeper system may pull shallower oxidized groundwater into deeper anoxic aquifers through nearby unused long-screen wells, thus releasing uranium from deeper aquifer sediments by oxidizing the uranium on exposed aquifer materials from U(IV) to U(VI)^45^. Poorly planned or operated uranium mining and milling facilities also may cause uranium to be released to groundwater systems, such as where mine wastewater or leach solution is allowed to infiltrate the subsurface^53^.

## Data and methods

As the objective of this investigation was to establish baseline groundwater conditions in the Grand Canyon region to monitor for changes in groundwater quality that may be the result of mining activities, data was compiled for, and additional samples were collected from, as many groundwater discharge locations as possible that could be accessed in the area. A particular focus was on establishing groundwater conditions at spring sites near historical, current, or planned breccia-pipe uranium mines, and at all groundwater wells in the area for which sampling permission could be obtained. The dataset for this assessment of uranium in groundwater in the Grand Canyon region was derived from both historical and recent (since 2009) USGS groundwater sample collection in the area, as well as samples collected by Grand Canyon National Park staff and analyzed by USGS. Uranium concentrations from groundwater samples were available from the USGS National Water Information System^54^ database from several USGS-led projects in the area over the years, with monitoring results since 2009 from ongoing sampling related to evaluating potential effects from breccia pipe uranium mining on regional water resources. Sampling results from many of these sites have been discussed in other studies of groundwater geochemistry in the area^20,26,55–57^. Uranium concentrations in groundwater also were available from a USGS–National Park Service (NPS) sampling partnership in 2016–2017, in which Grand Canyon National Park staff visiting spring locations in the park collected water samples for analyses by USGS laboratories. Analytical results from these samples are archived as a USGS data release^58^.

Groundwater samples were collected in acid rinsed sample containers at spring and well sites in the area, filtered through 0.45-µm capsule (Versapor acrylic copolymer membrane) or Luer–Lok syringe (polyethersulfone) filters, and preserved to pH < 2 with Ultrex grade nitric acid. Before the collection of groundwater samples from wells, the wells were first purged a minimum of three casing volumes and until stable properties (pH, water temperature, specific conductance, dissolved oxygen, and barometric pressure) were reached^59^. Field quality assurance measures included the collection, processing, and analysis of blanks and replicate samples. Filtered and preserved samples were analyzed for dissolved uranium by USGS laboratories using inductively coupled plasma–mass spectrometry (ICP–MS). Detection and reporting limits for ICP–MS analyses varied depending on instrument response to calibration and check standards over time. In this manuscript, results presented with “<” are less than the reporting limit for that ICP-MS run. Additional field property (e.g., pH, dissolved oxygen, alkalinity) data were available for some samples at some sites and are presented in Supplementary Table 1. Stability diagrams discussed in this manuscript and presented in the Supplementary Information were developed using Geochemist Workbench software.

## Results and discussion

To evaluate the precision of field and laboratory methods, 84 paired replicate samples were analyzed (Supplementary Fig. S3). Replicate analyses for dissolved uranium were comparable to environmental samples, with an average of 3% difference (difference/average) between the two. Replicates were available across a range of concentrations, with only the 10 to < 20 µg/L range having an average percentage difference greater than 3% (owing to a single 24% difference between environmental and replicate results of 14 and 11 µg/L, respectively). Dissolved uranium results were available for 27 field blanks, all with less-than-reporting limit concentrations except for a single reported value of 0.0015 µg/L. A single trip blank and equipment blank in the dataset also had less-than-reporting limit uranium concentrations. Four preservation blanks, whereby the acid used for sample preservation is analyzed on its own, all had reportable uranium concentrations, all ≤ 0.008 µg/L. Based on the results from the analyses of blank sample results, reported concentrations greater than 0.015 µg/L are unlikely to be affected by contamination.

Analytical results for uranium in groundwater in the Grand Canyon region were available for 573 samples collected from 206 sites by USGS and NPS scientists from September 1, 1981, to October 7, 2020 (Supplementary Table S1). Over 60% of the uranium samples for the area are from sampling since the 2009, when studies were undertaken to provide additional data on baseline groundwater quality in the region (Supplementary Fig. S4). Of the 206 groundwater sites for which dissolved uranium concentration data are available, 180 are spring sites and 26 are wells (Supplementary Table S1). Spring sampling locations are primarily along canyon walls and floors with wells located on plateaus above. Samples were collected from springs issuing from stratigraphic units above, within, and below the Permian strata that host uranium ore in breccia pipes in the area (Supplementary Table S1). Groundwater samples were collected from wells with depths ranging from 24 m to more than 1100 m (Supplementary Table S1).

**Figure 3:**
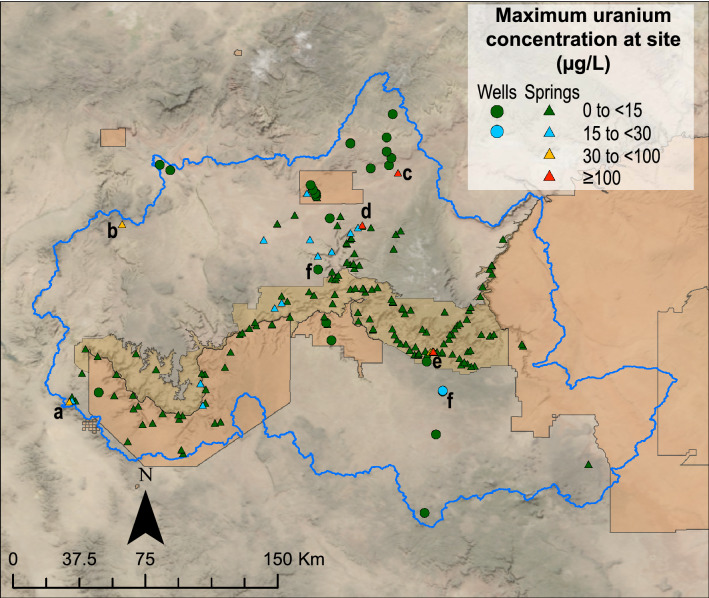
Map of maximum uranium concentration at 206 groundwater sites in the Grand Canyon region. Letters indicate sites that are discussed in the text. Map created in Esri ArcMap 10.8.1 (https://www.esri.com/). Basemap images from the public domain USGS National Map^11^.

**Figure 4:**
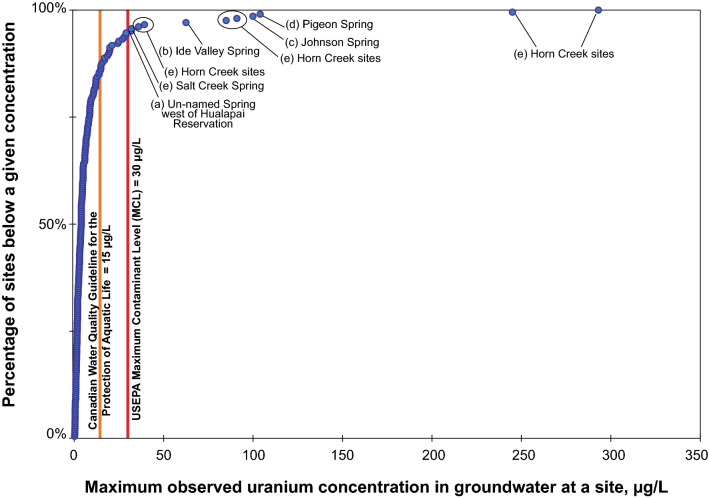
Distribution of the maximum concentration of uranium in groundwater samples from 206 sites in the Grand Canyon region. Letters indicate sites in Fig. 3 and are further discussed in the text.

The breadth of sample locations and uranium analytical results details are difficult to see at the scale presented in Fig. 3. Readers are invited to explore all results on the USGS Uranium in Groundwater in the Grand Canyon Region interactive map at https://webapps.usgs.gov/uraniummap/. Maximum uranium concentrations at groundwater sites in the region ranged from less than 1 µg/L at 23 sites (11%) to 100 µg/L or more at 4 sites (Supplementary Table S1, Fig. 3). Of the 206 groundwater sites sampled, 195 sites (95%) had maximum observed uranium concentrations less than the USEPA MCL of 30 µg/L for drinking water and 177 sites (86%) had uranium concentrations less than the Canadian benchmark for protection of aquatic life in freshwater of 15 µg/L^60^ (Supplementary Table S1; Fig. 4). Note that the United States does not have aquatic life benchmarks for uranium.

Of the 573 groundwater sampling events for which dissolved uranium was analyzed, pH data also were available for 385 (67%) of the samples (Supplementary Table S1). pH was circumneutral (6.5–7.5) in 197 of these samples, with pH values of 6.0 to 8.0 in 303 of these samples (Supplementary Table S1). Dissolved oxygen data were available from 331 of the 573 groundwater samples (Supplementary Table S1; Supplementary Fig. S5). In all but 17 of these samples (95%), groundwater is oxic, with dissolved oxygen concentrations equal to or greater than 0.5 mg/L^46^. As described previously, in oxic groundwater conditions uranium forms stable, readily soluble ionic or neutral complexes that are highly mobile^49^.

Of the 11 sites where uranium concentrations in groundwater were above the MCL, two sites (discussed next) were distant from mining locations and were thus unlikely to have been affected by mining activities. Located west of the Hualapai Reservation, spring B-28-16 09CCC (USGS site ID 354924114001200, labeled (a) in Fig. 3) issues from Precambrian granite, had a uranium concentration of 32 µg/L in 1993, and is 81 km from the closest breccia pipe uranium mine (Ridenour Mine). This spring is near the watershed divide that defines the study area for this investigation and likely has a local recharge source (tritium results of 21.0 and 20.0 pCi/L from samples collected in 1993 and 1994). Further investigation would be needed to identify the source of elevated uranium at this site. Ide Valley Spring (B-38-13 06BAD1, USGS site ID 364344113441701, labeled (b) in Fig. 3) issues from the Moenkopi Formation (which is above, or younger than, stratigraphic units in which uranium ore is found in breccia pipes in the area), had a maximum observed uranium concentration of 62.5 µg/L in 2010, and is 67 km from the nearest breccia pipe uranium mine (Chapel Mine). Elevated uranium concentrations at this spring are likely related to stratabound uranium deposits discussed in the introduction. Another spring in the study area with elevated uranium concentrations that are unlikely to be a result of breccia-pipe mining activities is Johnson Spring. Johnson Spring (A-42-01 31DDD, USGS site ID 365928112175201, labeled (c) in Fig. 3) had a maximum observed uranium concentration of 100 µg/L in 2010 and is somewhat closer to historical breccia-pipe uranium mines—between 35 and 50 km from three former breccia pipe uranium mines (Pigeon Mine, Kanab North Mine, and Hermit Mine). Johnson Spring, however, issues from the Shinarump Member of the Chinle Formation, which, like Ide Valley Spring, is above stratigraphic units in which uranium ore is found in breccia pipes in the area (Fig. 2). Additionally, the elevation of Johnson Spring is 40 m or more above the land surface elevation at the three closest mines. Similar to Ide Valley Spring, the elevated uranium concentrations at Johnson Spring are likely related to stratabound uranium deposits. The remaining 8 sites, Pigeon Spring and springs in and near Horn Creek (labeled as (d) and (e) in Fig. 3), will be discussed in more detail below.

### Pigeon Spring

**Figure 5:**
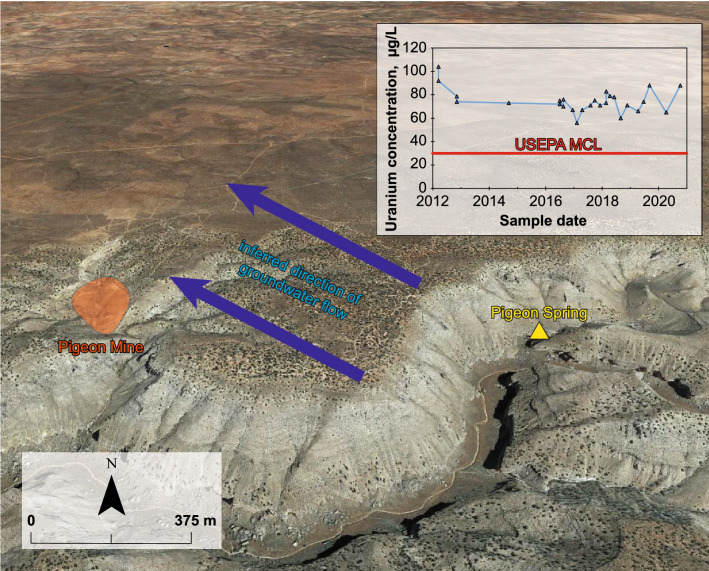
Map of Pigeon Spring and the former Pigeon Mine with uranium concentrations through time at the spring and inferred direction of groundwater flow from Beisner et al.^62^. Created in Adobe Illustrator 25.4.1 (https://www.adobe.com/products/illustrator.html). Oblique imagery from Google, 2021.

Pigeon Spring (B-38-02 04ACA1, USGS Site ID 364327112303101, (d) in Fig. 3) is located 1.7 km from the former Pigeon Mine uranium mine (Fig. 5). The Pigeon breccia pipe was mined from 1985 to 1990 with 2585 metric tons of uranium oxide (U_3_O_8_) produced at the mine^61^. The mine was reclaimed after it closed in 1990. Pigeon Spring discharges from perched groundwater within the Toroweap Formation (Fig. 2), most likely after having moved down through the Kaibab and Toroweap Formations^62^. USGS has been monitoring water chemistry at Pigeon Spring since 2012, and uranium concentrations at the site are the highest observed in the study area north of Grand Canyon—as high as 104 µg/L on March 15, 2012 (Fig. 5). Temporal changes in uranium concentrations, which may be due to fluctuations in recharge amount and seasonality, are apparent in the dataset, with concentrations averaging 73 µg/L and varying from 56 to 104 µg/L between 2012 and 2020^63^. Beisner et al.^62^ published a detailed description of water chemistry at Pigeon Spring and nearby springs and investigated the likelihood that the source of elevated uranium concentrations at the site was Pigeon Mine. Based on estimated direction of groundwater flow inferred from spring elevations in the area and comparison of uranium concentrations at Pigeon Spring to leaching experiments involving Pigeon Mine waste rock, ore, and nearby soil, Beisner et al.^62^ concluded that current evidence indicate it is unlikely that elevated uranium at Pigeon Spring is coming from the former Pigeon Mine. This conclusion is important for establishing expectations of naturally occurring concentrations of uranium (and other associated trace elements) in groundwater in the region. Ongoing monitoring at the site and samples from a planned nearby well will provide additional data on temporal changes in geochemistry in groundwater at the site and allow for further evaluation of the relationship between the mine and spring.

### Horn Creek area

**Figure 6:**
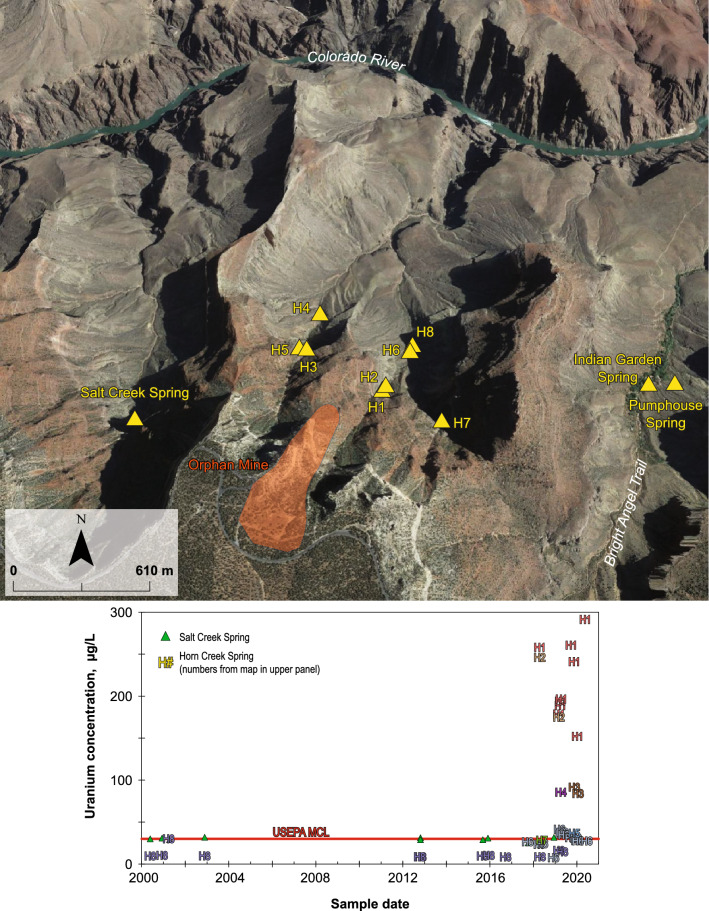
Map of former Orphan Mine uranium mine with location of nearby springs (upper panel) and time series chart of uranium concentrations in springs in the Horn and Salt Creek drainages (lower panel). Springs in the Horn Creek drainage are labeled from highest to lowest uranium concentration (H1, H2, etc.) in both panels. Created in Adobe Illustrator 25.4.1 (https://www.adobe.com/products/illustrator.html). Oblique imagery from Google, 2021.

The Horn Creek drainage within Grand Canyon National Park contains spring sites with the highest observed uranium concentrations in groundwater in the region, as high as 293 µg/L. This area is of interest to scientists and resource managers owing to its high uranium concentrations in groundwater and its proximity to the abandoned Orphan Lode Mine uranium mine (a.k.a. Orphan Mine, Fig. 6). The Orphan Mine was patented for copper in 1906, 26 months before President Theodore Roosevelt established Grand Canyon National Monument and 13 years before the establishment of Grand Canyon National Park by President Woodrow Wilson^64^. Uranium was found in the old workings of the copper mine in 1951 and uranium mining began in 1956^29^. In 1962, President John Kennedy signed a law permitting the mining operator to mine uranium ore in Grand Canyon National Park in an area adjacent to the Orphan claim, in exchange for title to the mine to be given to the U.S. Government in 25 years (in 1987)^29^. Between 1956 and 1969, when mining ceased at the site, 1932 metric tons of uranium oxide were produced at Orphan Mine, in addition to 3030 metric tons of copper^29^. After the mine closed, the U.S. Atomic Energy Commission estimated that 227 metric tons of uranium oxide remained in the mine^29^. As of 2021, the National Park Service continues to evaluate the site to determine if cleanup action is required.

Groundwater discharges from rock units and streambed alluvium at several locations in the Horn Creek drainage and uranium concentrations in groundwater vary greatly among these sites (Fig. 6). The highest uranium concentration in groundwater at Horn Creek, and the highest concentration observed at any groundwater site sampled by USGS in the region, is 293 µg/L from a sample on June 2, 2020, at site “Upper Horn Bedrock Spring” (H1 in Fig. 6). This spring issues from the Redwall–Muav aquifer at the canyon wall just downslope from the Orphan Mine. The first documented sampling of this site for uranium was in 2002 with reported uranium values as high as 400 µg/L^65^ (which was not part of a USGS study, and thus not included in the current dataset). Uranium concentrations at this spring have varied considerably since USGS began monitoring the site in 2018, with concentrations as low as 151 µg/L in January 2020, just 6 months before the high of 293 µg/L (Supplementary Table S1). Uranium concentration fluctuations may be due to changes in recharge amount and seasonality and is part of ongoing investigation. Observed pH values of 7.4–8.2, oxic dissolved oxygen concentrations of 8.2–10.1 mg/L, and the presence of bicarbonate from 148–154 mg/L (Supplementary Table S1) result in expected mobile uranium carbonate speciation (Supplementary Fig. S6). As the water flows down the Horn Creek drainage from the Upper Bedrock Spring the concentration remains high at a site about 60 m below the emergence of the water (H2 in Fig. 6) with observed uranium concentrations of 174 and 245 µg/L (Supplementary Table S1). Three springs in a separate headwater drainage of Horn Creek just to the west also exhibit elevated uranium concentrations, ranging from 36 to 91 µg/L (H3–H5 in Fig. 6). Another three springs sampled by USGS in the eastern headwater drainage had uranium concentrations mostly at or below the USEPA MCL (H6–H8 in Fig. 6), with concentrations as low as 14 µg/L in the latest sample (April 2019) at the most downslope site monitored in the drainage (H8).

A spring in the Salt Creek drainage, just to the west of Horn Creek, has observed uranium concentrations of 25–32 µg/L (Supplementary Table S1). Important water sources at the nearby Indian Gardens campground along the heavily trafficked Bright Angel Trail in Grand Canyon National Park have, to date, low uranium concentrations, with Indian Gardens spring sampled at 1.6–2.3 µg/L and Pumphouse spring sampled at 1.7–1.9 µg/L (Supplementary Table S1, Fig. 6). The USGS is currently conducting studies to establish the source of high uranium concentrations in Horn Creek area springs and to better understand spatial and temporal changes in uranium and other trace elements in the drainage^66^. Although the Orphan Mine is not an example of modern mining and reclamation practices, understanding the possible connection between the mine and Horn Creek springs would establish an important indicator of groundwater chemistry affected by a mine.

### Groundwater sampling at breccia-pipe uranium mine locations

**Figure 7:**
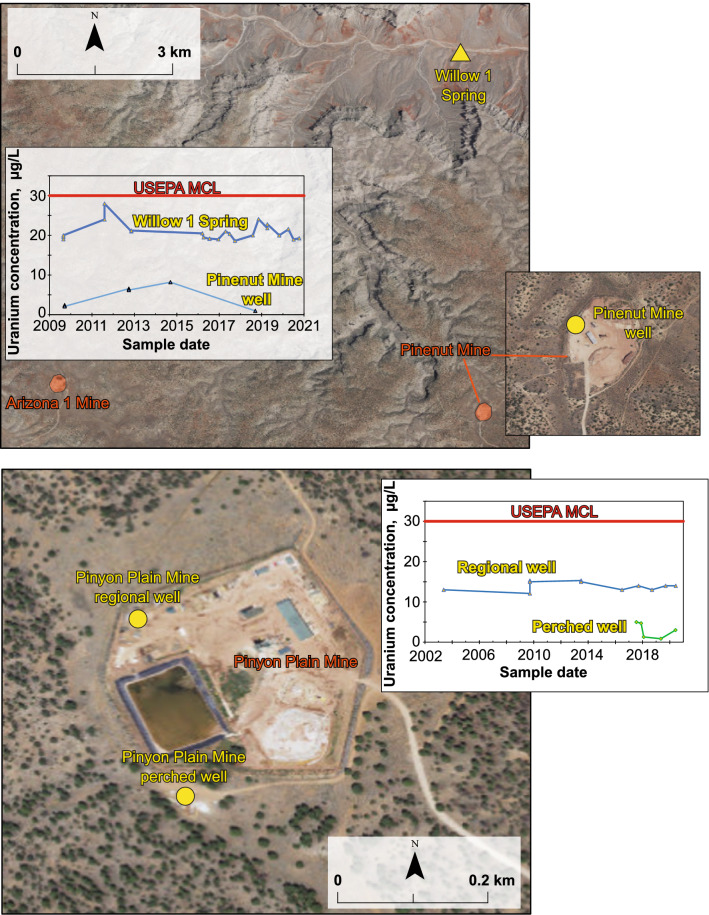
Groundwater sites monitored by USGS at and near breccia-pipe uranium mining locations in the Grand Canyon region including the Pinenut Mine (top panel) and the Pinyon Plain Mine (bottom panel). Map created in Esri ArcMap 10.8.1 (https://www.esri.com/). Basemap image from the public domain USGS National Map^11^.

The USGS samples three groundwater wells at two breccia-pipe uranium mine locations in the Grand Canyon area (locations labeled (f) in Fig. 3). The Pinenut Mine well (USGS Site ID 363003112440901) is located on the former Pinenut Mine site north of Grand Canyon and is used to meet operational needs at both the Pinenut and Arizona 1 (6.5 km from Pinenut) mines (Fig. 7). The Pinenut Mine was first mined in the 1980s, placed on standby from 1989 until 2013, and then was mined again until 2015 when the mine was closed and began reclamation^27^. The nearby Arizona 1 mine was developed in the early 1990s but did not begin producing ore until 2009^27^. Currently, Arizona 1 is in standby. The Pinenut Mine well was drilled in 1986 to a total depth of 975 m and is screened in the Mississippian-age Redwall Limestone. With cooperation from the mine operator, Energy Fuels, USGS has sampled the well on four occasions, the most recent in 2018 (Fig. 7). Uranium concentrations in the Pinenut Mine well have all been substantially less than the USEPA MCL, with a highest concentration of 6.5 µg/L in 2012 (Supplementary Table S1). A nearby spring, Willow 1 Spring (USGS Site ID 363357112440801), just 7 km from the Pinenut Mine, also is monitored by USGS. Uranium concentrations at Willow 1 Spring have ranged from 18.6 to 28 µg/L over the 18 sampling events since 2009 (Fig. 7, Supplementary Table S1).

At the Pinyon Plain Mine (formerly known as the Canyon Mine) south of Grand Canyon, the USGS monitors a well in the shallow groundwater at the site (perched well) and periodically samples a well in the deeper regional aquifer (Fig. 7). Uranium ore was identified at Pinyon Plain mine in 1983 and surface development of the mine site began in the 1990s but was paused until beginning again in 2012^27^. Mine shaft construction is complete, but as of 2020 no uranium ore has been mined from the site and the mine is in standby. The regional well at Pinyon Plain Mine (USGS Site ID 355308112054101) is located on the mine site and was drilled in 1986. The total depth of the regional well is 940 m and the well is screened from 788 to 902 m, over the Redwall–Muav aquifer. The regional well is sampled periodically by USGS with cooperation from the mine operator, Energy Fuels. A shallow well was installed in 2017 just outside the mine site to monitor water quality and groundwater levels in the perched groundwater near the mine. It is hypothesized that any effects from mining activities on groundwater would first be observed in the shallow system closest to the mine shaft and ore deposits. The perched groundwater monitoring well (USGS Site ID 355254112054901) was drilled to a depth of 354 m and screened from 340 to 346 m, over the bottom portion of the Coconino Sandstone.

Uranium concentrations in groundwater at Pinyon Plain Mine have ranged 12.1–15.3 µg/L in the deeper regional well and 0.9–5 µg/L in the shallower perched well (Fig. 7, Supplementary Table S1). Water in both the perched (% modern C-14 from 17.39 to 18.08; delta C-13/C-12 from − 8.13 to − 6.70 per mil) and regional (% modern C-14 from 13.82 to 16.8; delta C-13/C-12 from − 7.69 to − 6.94 per mil) wells have radiocarbon ages that indicate the mean age of the water is greater than 10,000 years old^67^. Anoxic conditions in perched groundwater at the site (dissolved oxygen ≤ 0.1 mg/L during all sampling events) indicates limited mobility of dissolved uranium in the shallow groundwater system were there to be an effect from mining activities. However, alkalinity of 230–243 mg/L (as CaCO_3_) and pH of 7.4–7.6 in the perched groundwater would promote uranium complex formation and increase the mobility of uranium in groundwater^47^ (Supplementary Fig. S7). In the deeper regional well, observed pH values of 7.0–7.7, oxic conditions evidenced by dissolved oxygen concentrations of 1.3–6.8 mg/L, and the presence of bicarbonate of 244–266 mg/L (Supplementary Table S1) result in expected mobile uranium carbonate speciation (Supplementary Fig. S8). Having established baseline water chemistry conditions in both the shallow and deeper groundwater systems at the site prior to commencement of ore production, continued monitoring throughout the mine’s lifecycle and after reclamation will allow for the assessment of changes that may result from mining activities.

### Comparison of uranium concentrations in groundwater in the Grand Canyon region to results from other studies

Data and information in other published studies can provide perspective on the range of uranium concentrations found in groundwater in the Grand Canyon area, a selection of which are summarized here. As described above, in 559 drinking water wells sampled in the southwestern United States, uranium concentrations exceeded the MCL in 7.3% of samples, with wells in the Central Valley aquifer system of California exceeding the MCL in 22% of samples^46^. Follow-up investigations on the relatively high concentrations of uranium in Central Valley groundwater found concentrations in the eastern part of the San Joaquin basin of nearly 500 μg/L^40^. The geologic source of the elevated levels of uranium in groundwater in the Central Valley is thought to be uranium in glacially eroded granitic rocks from high elevations in the Sierra Nevada mountains. Sediments derived from these rocks are leached by high bicarbonate recharge, primarily from irrigated agriculture, mobilizing uranium in groundwater^40,52,68^. Blake et al.^69^ report dissolved uranium concentrations of 67–169 μg/L in spring and seep water samples near an abandoned uranium mine site in a northeastern Arizona Native American community. Studies of uranium in groundwater in northeastern Washington state reported that uranium was detected (> 1 μg/L) in samples from 60% of the 1742 wells sampled, with samples from 87 wells (5%) exceeding the uranium MCL^70,71^. Uranium concentrations in spring and well samples in the Washington state study ranged from < 1 to 88,600 μg/L with a median of 1.4 μg/L. The highest concentrations were associated with monitoring wells near the inactive Midnite Mine uranium mine site located on the Spokane Tribe of the Spokane Reservation^71,72^. The mine was operated from 1955 until 1981 and several potential sources of uranium remain on site including open pits, waste rock piles, and ore stockpiles^72^.

Selected examples from international studies of uranium in groundwater include both naturally occurring and mining-affected locations. Coyte et al.^73^ presented data on uranium concentrations in 324 wells sampled in the states of Rajasthan and Gujarat in western India. The World Health Organization (WHO) provisional health guideline of 30 μg/L was exceeded in 80 (25%) of the wells sampled, with 54 (17%) wells exceeding 50 μg/L and 20 (6%) wells exceeding 100 μg/L (maximum uranium concentration of 320 μg/L). Naturally occurring uranium in crystalline basement source rocks and alluvial aquifer material that promotes the formation of soluble uranyl carbonate complexes are thought to play a role in elevated uranium concentrations in wells in western India^73^. Haakonde et al.^53^ presented uranium concentrations in drinking water sources in the vicinity of a uranium mine in southern Zambia. Uranium concentrations in 65 borehole water samples ranged from 17 to 263 μg/L and concentrations in 41 shallow wells ranged from 7 to 199 μg/L^53^. Haakonde et al.^53^ concluded that elevated uranium concentrations in groundwater samples are likely due to groundwater contamination from the mine’s wastewater dam. Results from these studies show that uranium concentrations in groundwater can vary greatly and that elevated uranium concentrations in groundwater may be found in both mining-affected and non-mining settings.

## Conclusions

Lack of scientific data on potential effects of uranium mining activities on cultural, biological, and water resources in the area led to the withdrawal of over 4000 km^2^ of Federal land in the Grand Canyon region from new uranium mining activities until 2032, subject to valid existing rights. This manuscript presents results for the investigation of uranium in groundwater in the Grand Canyon region. Analytical results for uranium in groundwater in the Grand Canyon region were available for 573 samples collected from 180 spring sites and 26 wells from September 1, 1981, to October 7, 2020. Samples were collected from springs issuing from stratigraphic units above, within, and below the Permian strata that hosts uranium ore in breccia pipes in the area. Maximum uranium concentrations at groundwater sites in the region ranged from less than 1 µg/L at 23 sites (11%) to 100 µg/L or more at four sites. Of the 206 groundwater sites sampled, 195 sites (95%) had maximum observed uranium concentrations less than the USEPA MCL of 30 µg/L for drinking water and 177 sites (86%) had uranium concentrations less than the 15 µg/L Canadian benchmark for protection of aquatic life in freshwater. Of the 11 sites where uranium concentrations in groundwater were above the MCL, two springs were > 65 km from mining locations and a third spring discharged at an altitude of 40 m or more above the land surface elevation at the three closest mines. The remaining eight sites with above-MCL uranium concentrations were in proximity to former Pigeon and Orphan mine sites. A previous detailed investigation of the groundwater system near Pigeon Spring concluded that evidence points to a natural source, and not mining activities, as the cause of elevated uranium concentrations at the spring. The highest uranium concentrations in groundwater in the study area were observed at spring sites downslope from the abandoned Orphan Mine within Grand Canyon National Park. Ongoing studies are investigating the potential link between the mine and the groundwater chemistry at the springs.

Results from other published studies provide context for the range of uranium concentrations observed in groundwater in the Grand Canyon area. Groundwater sampling results from the Central Valley in California and states in western India indicate elevated (> 100 μg/L) uranium concentrations may be observed in groundwater when non-mining anthropogenic activities (i.e., irrigation and well development) mobilize natural sources of uranium. While no conclusive effects from breccia-pipe mining activities on uranium concentrations in groundwater samples collected to date (2021) in the Grand Canyon region can be confirmed (although the Horn Creek/Orphan Mine investigation is ongoing), the timing of potential effects may take many years to reach groundwater discharge locations. The establishment of baseline groundwater quality is an important first step in monitoring for change in water chemistry throughout the mining lifecycle and beyond to ensure the health of these critical groundwater resources.

## Supplementary Information

Supplementary Information.

Supplementary Table S1.

## Data Availability

The datasets generated and analyzed during the current study are available at the U.S. Geological Survey National Water Information System^54^ and through a USGS data release^58^. All data discussed in this manuscript are included in the published Supplementary Information files.
