# Supplementary material for: An assessment of uranium in groundwater in the Grand Canyon region
Source: Sci Rep. 2021 Nov 16;11:22157. doi: 10.1038/s41598-021-01621-8 (PMC8595346; doi:10.1038/s41598-021-01621-8)
Supplement: Supplementary file 1 — Supplementary Information. [file 41598_2021_1621_MOESM1_ESM.pdf]

SCIENTIFIC REPORTS
natureresearch

Supplementary Information for

An Assessment of Uranium in Groundwater in the Grand Canyon Region

Fred D Tillman[1*], Kimberly R. Beisner[2], Jessica R. Anderson[3], and Joel A. Unema[3]

[1]U.S. Geological Survey, Arizona Water Science Center, Tucson, Arizona, USA; email: ftillman@usgs.gov
[2]U.S. Geological Survey, New Mexico Water Science Center, Albuquerque, New Mexico, USA
[3]U.S. Geological Survey, Arizona Water Science Center, Flagstaff, Arizona, USA

*Corresponding author

This Supplementary Information provides additional material relevant to the investigation of uranium concentrations in groundwater in the Grand Canyon region. This material includes:

- Figure S1. Comparison of the U.S. Environmental Protection Agency Maximum Contaminant Level for metals commonly associated with mineralized breccia pipes in the Grand Canyon region.
- Figure S2. Map of the estimated mean surface concentration of uranium from airborne gamma-ray spectrometry.
- Figure S3. Box plots of percentage difference (difference/mean) results for replicate samples of uranium concentrations in groundwater.
- Figure S4. Histogram of number of groundwater sites sampled for uranium in a given year (left vertical axis) and cumulative percentage of uranium samples for all years (right vertical axis).
- Figure S5. Available dissolved oxygen and pH results for groundwater samples in the Grand Canyon region for which dissolved uranium was analyzed.
- Figure S6. Activity diagram showing predominance of uranium species over a range of pH and dissolved oxygen conditions. This diagram was constructed using the chemistry of groundwater discharging from the top of the Horn Creek drainage with the following concentrations: U (257 µg/L), HCO_3^- (148 mg/L), and SO_4^{2-} (309 mg/L), where HCO_3^-

and SO_4^{2-} were set to speciate over both diagram axes (dashed lines indicate the speciation areas for HCO_3^- and SO_4^{2-}.

- Figure S7. Activity diagram showing predominance of uranium species over a range of pH and dissolved oxygen conditions. This diagram was constructed using the water chemistry from the Pinyon Plain Mine perched groundwater well with the following concentrations U (1.3 μg/L), HCO_3^- (287 mg/L), and SO_4^{2-} (59 mg/L), where HCO_3^- and SO_4^{2-} were set to speciate over both diagram axes (dashed lines indicate the speciation areas for HCO_3^- and SO_4^{2-}.
- Figure S8. Activity diagram showing predominance of uranium species over a range of pH and dissolved oxygen conditions. This diagram was constructed using the water chemistry from the Pinyon Plain Mine regional groundwater well with the following concentrations U (13 μg/L), HCO_3^- (264 mg/L), and SO_4^{2-} (18 mg/L), where HCO_3^- and SO_4^{2-} were set to speciate over both diagram axes (dashed lines indicate the speciation areas for HCO_3^- and SO_4^{2-}.
- Table S1. Analytical results for uranium concentrations in groundwater samples from the Grand Canyon region (separate Excel file).

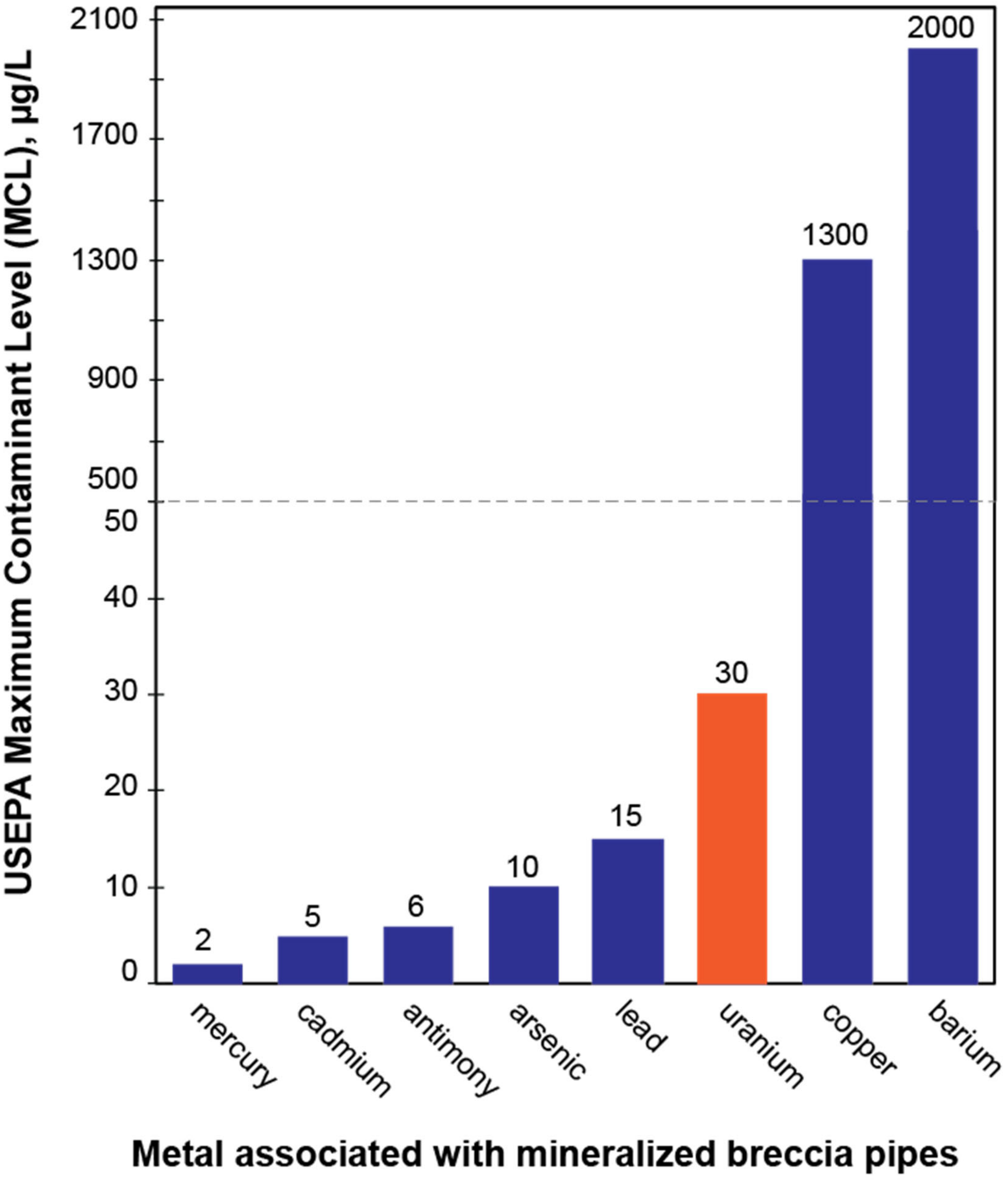

Figure S1. Comparison of the U.S. Environmental Protection Agency Maximum Contaminant Level[1] for metals commonly associated with mineralized breccia pipes in the Grand Canyon region. Note discontinuous vertical axis.

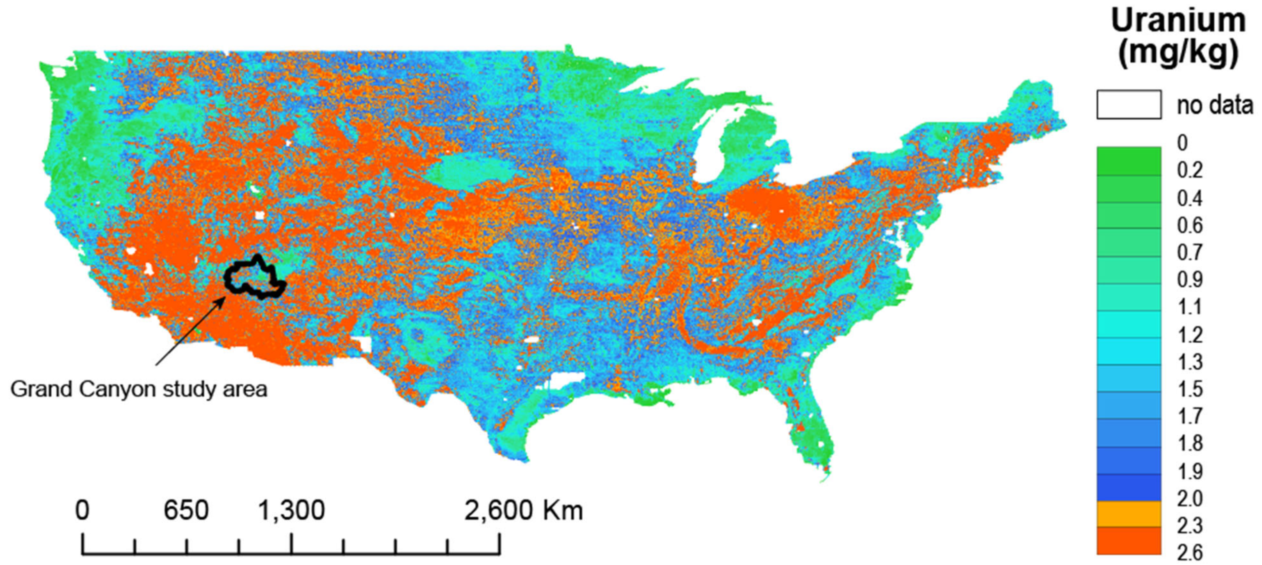

Figure S2. Map of the estimated mean surface concentration of uranium in the continental United States from airborne gamma-ray spectrometry[2] (AGRS).

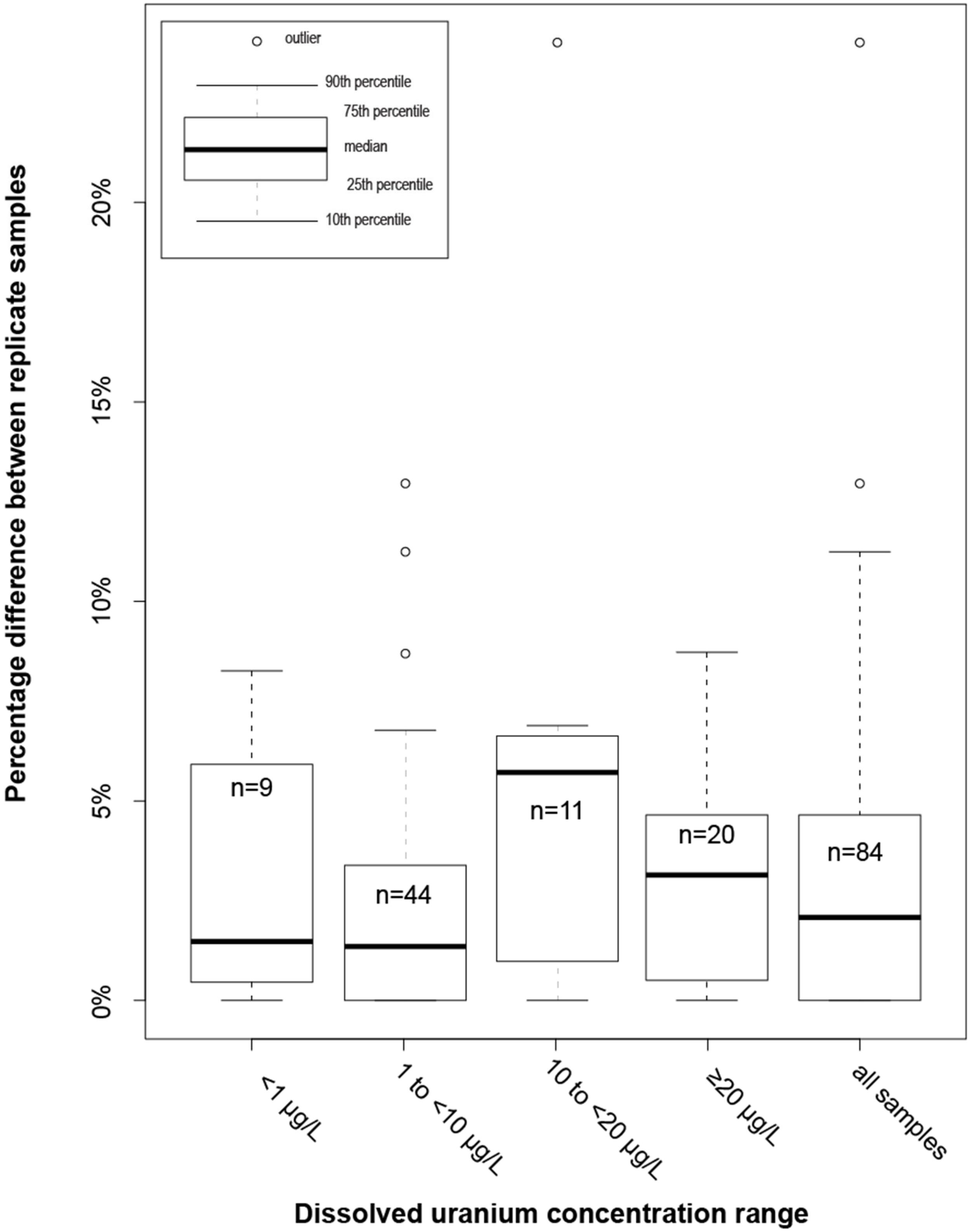

Figure S3. Box plots of percentage difference (difference/mean) results for replicate samples of uranium concentrations in groundwater.

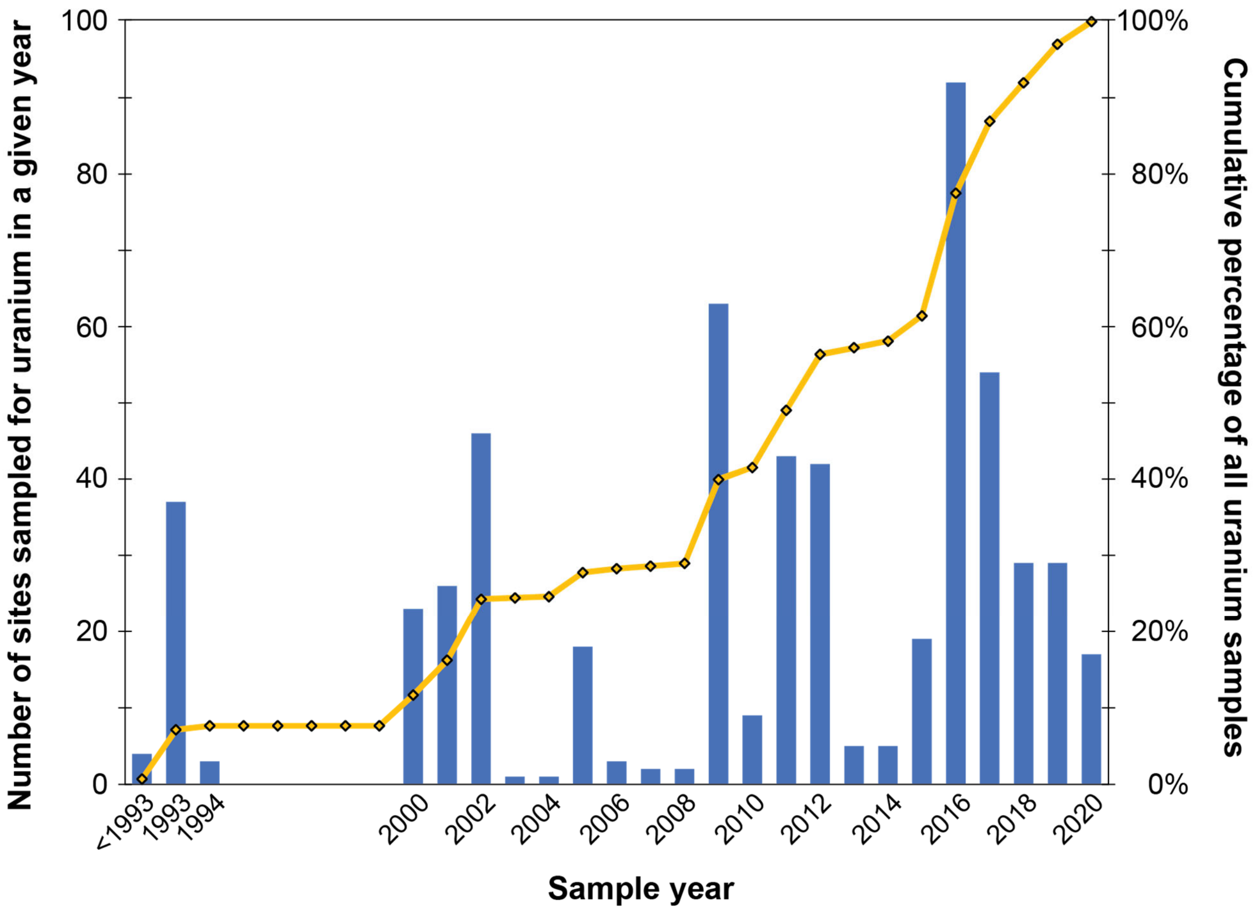

Figure S4. Histogram of number of groundwater sites sampled for uranium in a given year (left vertical axis) and cumulative percentage of uranium samples for all years (right vertical axis).

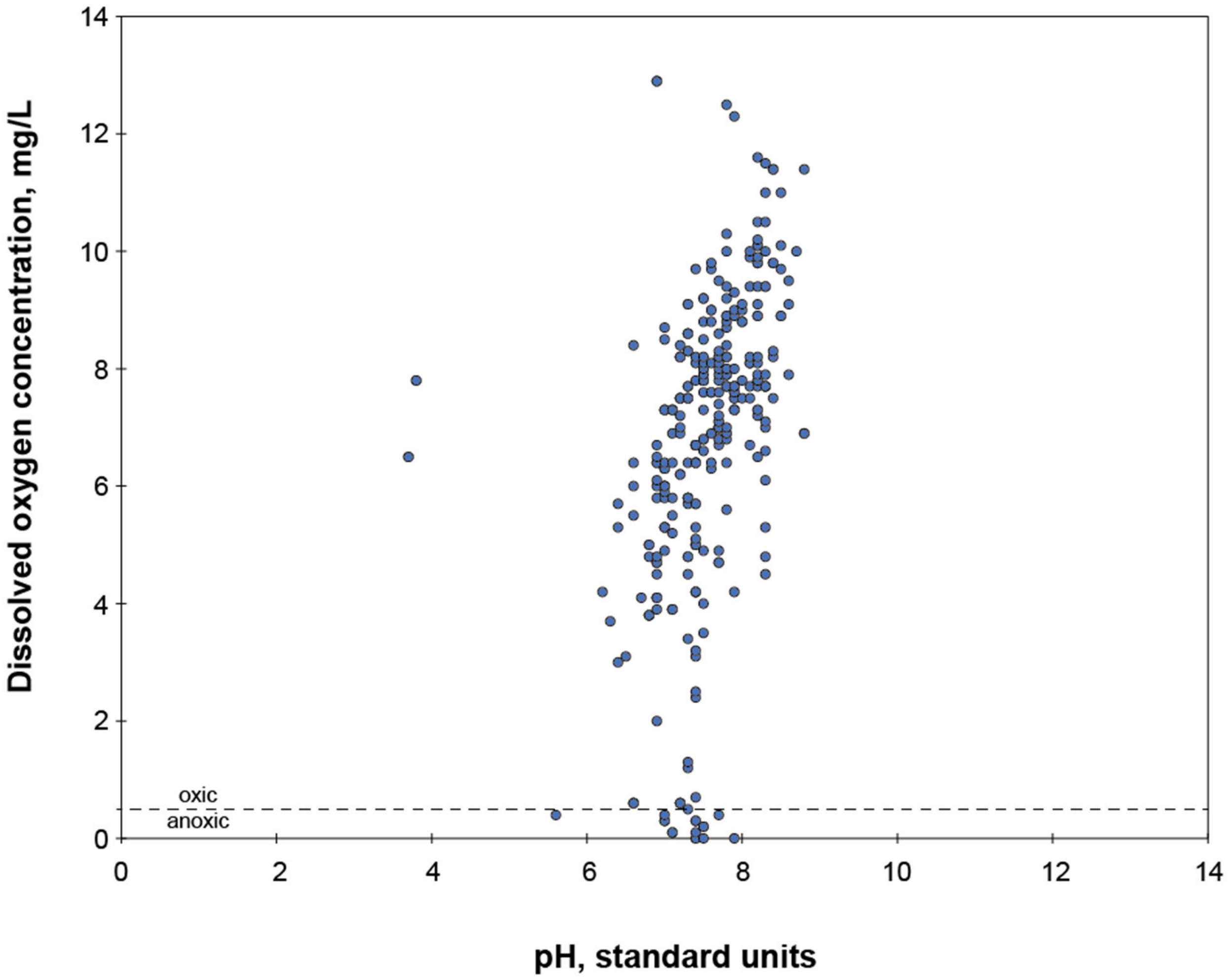

Figure S5. Available dissolved oxygen and pH results for groundwater samples in the Grand Canyon region for which dissolved uranium was analyzed.

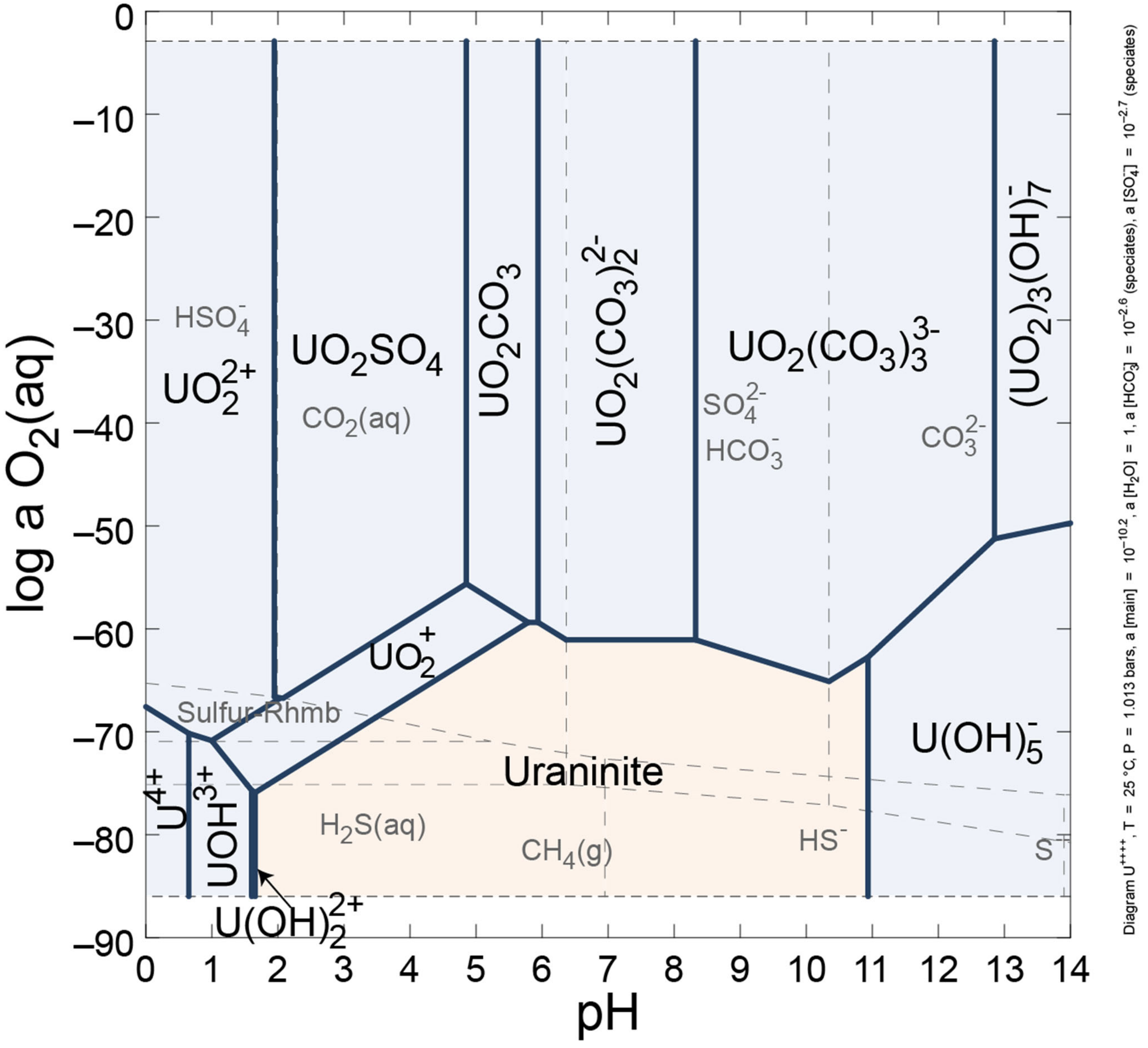

Figure S6. Activity diagram showing predominance of uranium species over a range of pH and dissolved oxygen conditions. This diagram was constructed using the chemistry of groundwater discharging from the top of the Horn Creek drainage with the following concentrations U (257 µg/L), HCO_3^- (148 mg/L), and SO_4^{2-} (309 mg/L), where HCO_3^- and SO_4^{2-} were set to speciate over both diagram axes (dashed lines indicate the speciation areas for HCO_3^- and SO_4^{2-}).

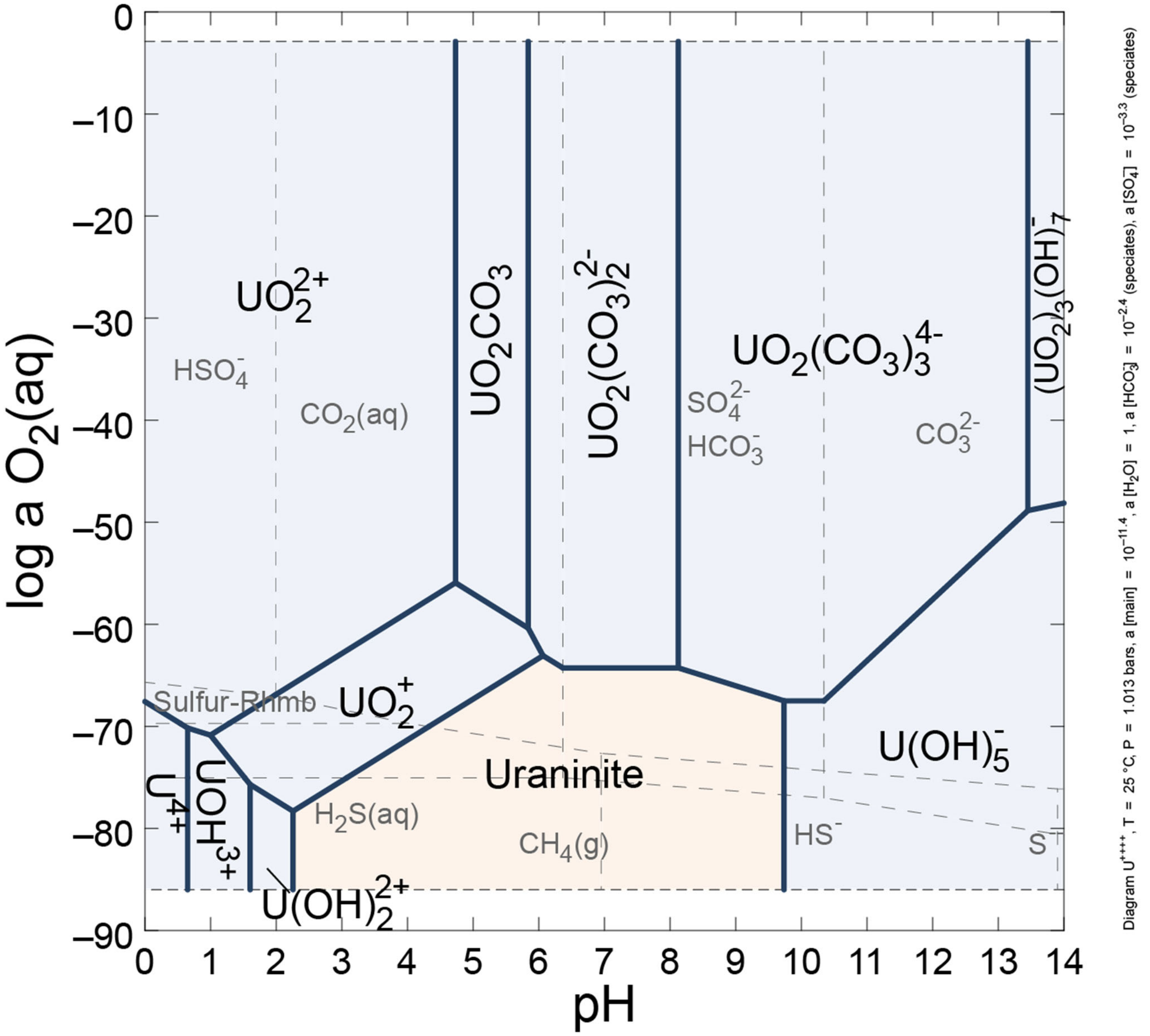

Figure S7. Activity diagram showing predominance of uranium species over a range of pH and dissolved oxygen conditions. This diagram was constructed using the water chemistry from the Pinyon Plain Mine perched groundwater well with the following concentrations U (1.3 µg/L), HCO_3^- (287 mg/L), and SO_4^{2-} (59 mg/L), where HCO_3^- and SO_4^{2-} were set to speciate over both diagram axes (dashed lines indicate the speciation areas for HCO_3^- and SO_4^{2-}).

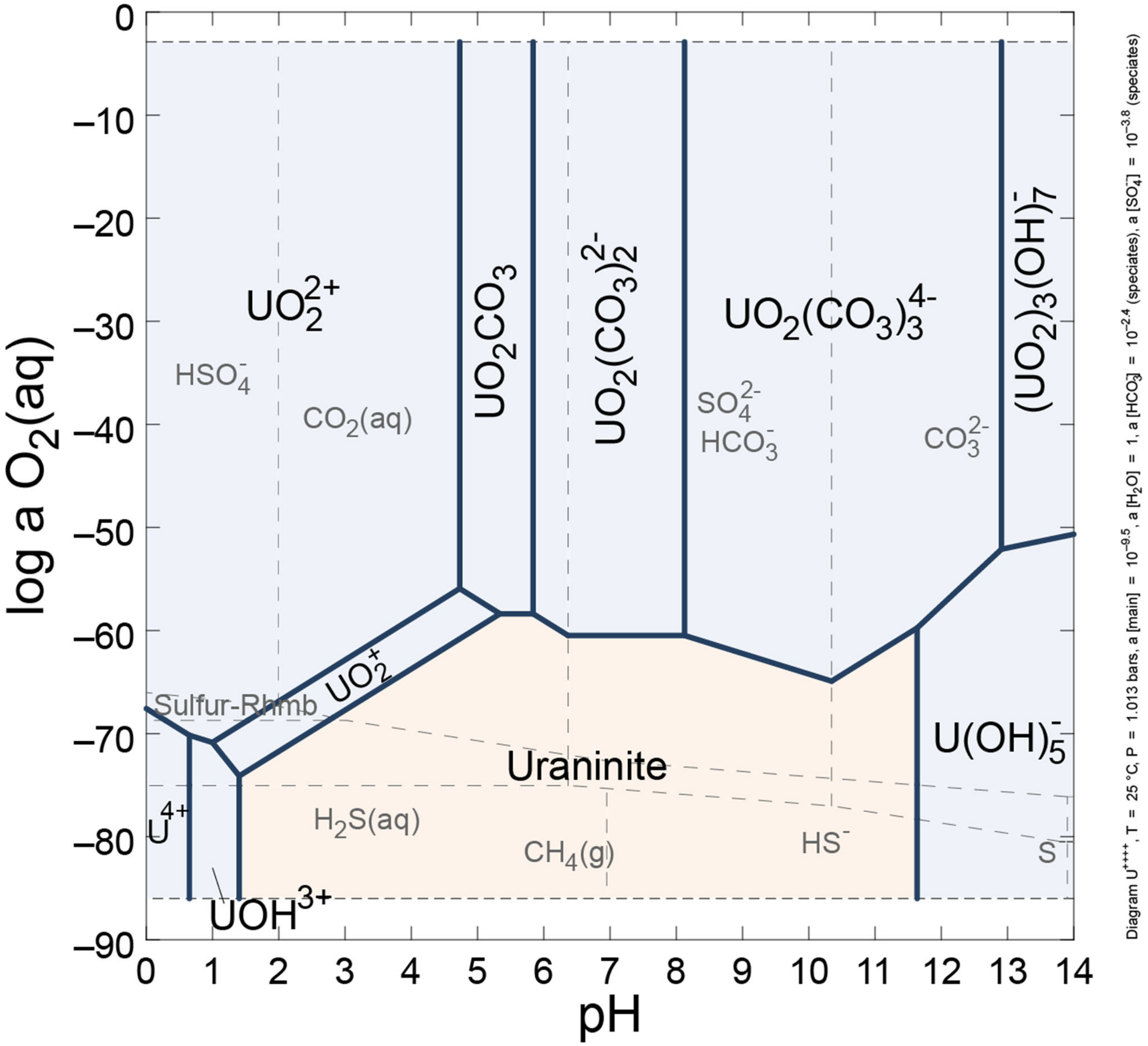

Figure S8. Activity diagram showing predominance of uranium species over a range of pH and dissolved oxygen conditions. This diagram was constructed using the water chemistry from the Pinyon Plain Mine regional groundwater well with the following concentrations U (13 µg/L), HCO_3^- (264 mg/L), and SO_4^{2-} (18 mg/L), where HCO_3^- and SO_4^{2-} were set to speciate over both diagram axes (dashed lines indicate the speciation areas for HCO_3^- and SO_4^{2-}).

References

1. U.S. Environmental Protection Agency. Drinking water contaminants. *US Environ. Protect. Agency database.* https://www.epa.gov/ground-water-and-drinking-water/national-primary-drinking-water-regulations. Accessed 17 Feb 2021 (2021).
2. Duval, J. S., Carson, J. M., Holman, P. B. & Darnley, A. G., Terrestrial radioactivity and gamma-ray exposure in the United States and Canada. *US Geol. Surv. Open-File Rep.* **2005–1413**. https://pubs.usgs.gov/of/2005/1413/ (2005).
